# The association between body fat percentage and self-reported depression in the United Arab Emirates

**DOI:** 10.1093/eurpub/ckac131.133

**Published:** 2022-10-25

**Authors:** M Al Balushi, A Ahmad, S Javaid, L Ahmed, F Al Maskari, A Abdulle, R Ali

**Affiliations:** 1 Public Health Research Center, New York University Abu Dhabi, Abu Dhabi, United Arab Emirates; 2 Institute of Public Health, United Arab Emirates University, Abu Dhabi, United Arab Emirates; 3 Department of Mental Health, United Arab Emirates University, Abu Dhabi, United Arab Emirates

## Abstract

**Background:**

The United Arab Emirates Healthy Future Study (UAEHFS) is one of the first large prospective cohort studies in the region which examines causes and risk factors for chronic diseases among adult UAE nationals. The aim of this study was to explore the relationship between body fat percentage (BF%) and the eight-item Patient Health Questionnaire (PHQ-8) as a screening instrument for depression among the UAEHFS pilot study participants.

**Methods:**

We analyzed the UAEHFS pilot data to investigate the association between BF% and PHQ-8 adjusted for age and gender. We used multivariate logistic ordinal regression model. To impute missing values, 100 multiple imputations (MI) were performed using multivariate imputation of classification and regression tree. The statistical analysis was performed using R Statistical Software (version 4.2.0)

**Results:**

Out of 517 participants, data from 487 (94.2%) were analyzed after excluding participants who didn't fill out the questionnaires. The median age was 30 years (Interquartile Range: 23 - 38). There were more males (67.8%) than females in the UAEHF pilot data. Approximately, 64 (13.1%) of the participant reported depression. The prevalence of obesity was 35.2% in this study population. The estimated odds ratio of BF% from the fitted multivariate logistic ordinal regression model was OR = 1.046 (95% CI: 1.012-1.08), and OR = 1.03 (95% CI: 1.003-1.057) for the omitted data, and MI (sensitivity analysis) respectively.

**Conclusions:**

High body fat percentage was statistically significantly associated with high risk of reporting depression. Additional research is needed, using the main UAEHFS data (after recruitment is complete), to further investigate the association between body fat percentage and depression.

**Key messages:**

• Our results can help contribute to the knowledge based on current and potential population mental health in the UAE and Gulf Region.

• The main finding of this study that excess body fat is associated with an increased risk of developing depression and vice versa; thus, this could add to the future direction of mental health research.

